# Factors Influencing Bacterial Diversity and Community Composition in Municipal Drinking Waters in the Ohio River Basin, USA

**DOI:** 10.1371/journal.pone.0157966

**Published:** 2016-06-30

**Authors:** Lee F. Stanish, Natalie M. Hull, Charles E. Robertson, J. Kirk Harris, Mark J. Stevens, John R. Spear, Norman R. Pace

**Affiliations:** 1 Department of Molecular, Cellular, and Developmental Biology, University of Colorado, Boulder, CO, United States of America; 2 Department of Pediatrics, University of Colorado Anschutz Medical Campus, Aurora, CO, United States of America; 3 Department of Civil and Environmental Engineering, Colorado School of Mines, Golden, CO, United States of America; Loyola University Chicago, UNITED STATES

## Abstract

The composition and metabolic activities of microbes in drinking water distribution systems can affect water quality and distribution system integrity. In order to understand regional variations in drinking water microbiology in the upper Ohio River watershed, the chemical and microbiological constituents of 17 municipal distribution systems were assessed. While sporadic variations were observed, the microbial diversity was generally dominated by fewer than 10 taxa, and was driven by the amount of disinfectant residual in the water. Overall, *Mycobacterium* spp. (*Actinobacteria*), MLE1-12 (phylum *Cyanobacteria*), *Methylobacterium* spp., and sphingomonads were the dominant taxa. Shifts in community composition from *Alphaproteobacteria* and *Betaproteobacteria* to *Firmicutes* and *Gammaproteobacteria* were associated with higher residual chlorine. Alpha- and beta-diversity were higher in systems with higher chlorine loads, which may reflect changes in the ecological processes structuring the communities under different levels of oxidative stress. These results expand the assessment of microbial diversity in municipal distribution systems and demonstrate the value of considering ecological theory to understand the processes controlling microbial makeup. Such understanding may inform the management of municipal drinking water resources.

## Introduction

Microbes are ubiquitous components of drinking water. From source to tap, physical, chemical, and biological processes can influence the microbial composition, with potential effects on water quality and safety. While maintaining a safe and reliable drinking water supply is of critical importance, relatively few potentially pathogenic microbes are acknowledged and even fewer are regulated. In the U.S. *E*. *coli* and fecal coliforms are well regulated, but numerous unregulated opportunistic pathogens such as *Mycobacterium* spp. [1–4] and *Legionella* spp. [5–7] can become abundant in distribution systems and thereby provide potential sources of disease [8–10]. There is strong evidence, for instance, that household plumbing can increase the risk of contracting nontuberculous mycobacterial disease [11,12]. Clearly, a more holistic approach to characterizing the microbiology of distribution systems is critical to ensure continued water safety and quality and inform future decisions in the face of expanding population densities.

Municipal drinking water distribution systems (DWDSs) in the U.S. consist of more than 1 million miles of underground pipes (12) fed by waters of highly variable quality. DWDSs typically present highly selective environments: dark, low in nutrients and, in the U.S., strongly oxidizing due to chlorine disinfection. Despite the extreme nature of the DWDS environment, microbial life persists and can thrive. Biofilms in particular can support microbial growth and activity, as they shield inhabitants from disinfectant [13,14], trap scarce nutrients [15] and provide for development of stable communities [16]. Advancements in detection and analysis of microbes have uncovered general patterns in the microbial ecology of DWDSs, and while considerable variability in physical and chemical properties exist, some commonalities are emerging [2,17,18]. Previous research has shown that many factors including plant design [19], chlorine disinfection [20,21], source water [2,19], water retention time [22], hydraulic regime [23], and water chemistry [2,24] can influence microbial communities. Given such a wide range of external factors, the relative importance of each individually is usually unclear and may vary in unknown and unpredictable ways. The considerable variability in microbial compositions of different systems and even different taps in the same network is a striking feature of municipal DWDSs [2].

Much of what is known about DWDS microbiology is derived from studies of single municipalities or simulated pipe systems in laboratory experiments, and substantial variability has been seen [3,21,25,26]. This limits our ability to identify patterns and to predict processes on a broader scale, and even limits our knowledge of the range of microbial diversity that inhabits DWDSs. Consequently, regional and culture-independent surveys are necessary to provide insights into larger biogeographic and geologic patterns that otherwise are difficult to discern. A previous study in chlorinated DWDSs found variations in microbial taxa that related to source water, while other taxa covaried with nitrate concentrations [2]. This suggests that certain taxa may act as indicator species for monitoring DWDS integrity and microbiological quality. A source-to-tap study in non-chlorinated DWDSs found that the water treatment plant structured microbial assemblages more than seasonal variability [19]. Both studies showed considerable variation in the composition and abundances of microbiology across municipalities. Additional such surveys are needed to flesh-out the inventory of DWDS bacterial diversity and identify commonalities, and to explore regional variations. Findings from such studies could contribute to improved drinking water monitoring technology and regulations and potentially improve public health.

The goal of the current study was to examine the drivers of bacterial composition and diversity in finished tap waters in a relatively under-studied region of the U.S. The microbiology and chemistry of tap waters were determined for 17 DWDSs in the central-eastern U.S., including Ohio, Pennsylvania, West Virginia, and Kentucky. The survey was conducted on small to medium-sized DWDSs, which are poorly studied but represent the vast majority of municipal DWDSs in the U.S.: 98% serve communities of less than 50,000 people [27]. We sought to determine: what are the major factors influencing bacterial communities? Are there commonalities in bacterial taxa across DWDSs, and could these common suites of bacteria indicate water quality status? Finally, how does variation in the physical and chemical environment of the DWDS influence tap water microbiota?

## Methods

### Sample sites

**Fig 1:**
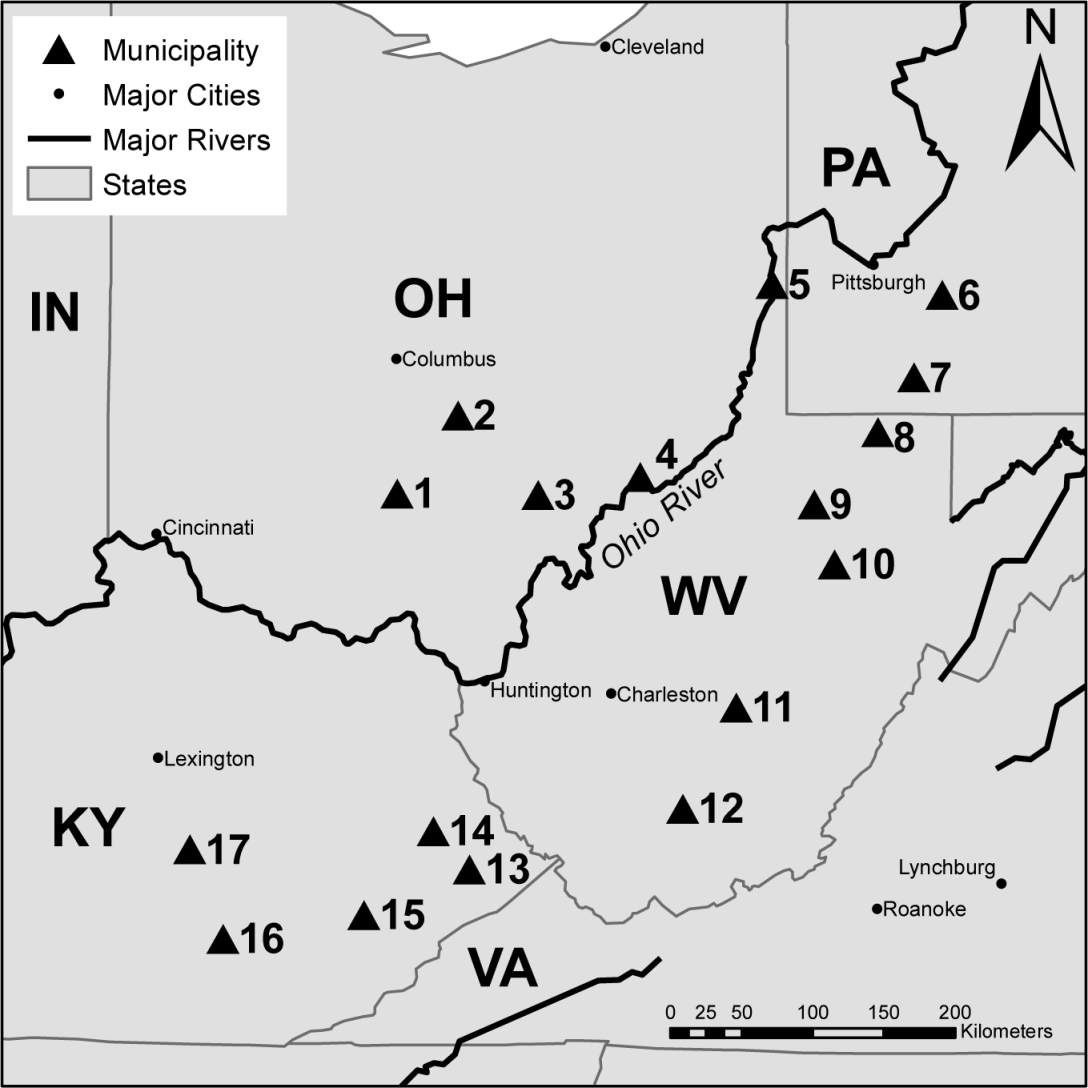
Map of study region. Upper Ohio River region showing the locations of municipalities where tap waters were sampled. Triangles denote municipality locale.

A sampling campaign was conducted during July of 2013 in 17 moderate-sized municipalities located within the headwaters of the Ohio River Basin (Fig 1 and S1 Table). The municipalities ranged in size from approximately 3,000 to 50,000 customers. Five sample locations were randomly selected within each municipality with an effort to broadly represent the areal extent of the DWDS. Water samples were collected from taps located in businesses and public buildings with open, public access. Most sample locations were on public land and did not have specific permission requirements. The privately owned buildings were establishments that served the broader public, and were sampled during business hours with the permission of store managers. Sample taps were anonymized in order to protect the identities of tap owners.

### Sample collection

Distribution system waters were sampled from cold-water taps by flushing the tap to clear out premise plumbing water. Sampling proceeded after the water temperature measured by a hand-held meter had a stable reading for at least one minute. Free and total/combined chlorine (Cl) were measured on-site using a hand-held meter (Hannah Instruments, model HI-96711, Smithfield, RI), which was calibrated prior to and during the field campaign. Water temperature (WT), dissolved oxygen (DO), pH, and salinity were measured on-site using a hand-held multi-probe (Hannah Instruments, model HI-9828). For ion analyses, 50 mL of water was filtered on-site using a cellulose acetate filter of 0.45 μm pore size (Advantec MFS, Inc., Dublin, CA), and filtrates were stored on ice for up to 7 days prior to being shipped to the Colorado School of Mines for analysis. Ion analysis was conducted using a Dionex ICS-90 ion chromatography system running an AS14A (4 x 250 mm) column. Major cations were measured using a Perkin-Elmer Optima 5300 DV Inductively Coupled Plasma Optical Emission Spectrometer (ICP-OES). Water for total organic carbon (TOC) analysis was collected on-site in acid-washed, pre-combusted 125 mL glass bottles and stored on ice. Samples were acidified within 8 hours of collection with concentrated hydrochloric acid to achieve a pH of < 2, and remained on ice until analysis took place at the University of Kentucky in Lexington. TOC was measured using a Shimadzu TOC analyzer (TOC-5000A). Water samples for metals analysis were collected in sterile, 50 mL conical tubes (Grenier), stored on ice, acidified within 8 hours with concentrated nitric acid to pH <2 and kept on ice until analysis. Metals were measured using a Vista-MPX CCD-Simultaneous ICP-OES (Varian Analytical Instruments) at the University of Kentucky. Water for microbial cell counts was collected in sterile 50 mL conical tubes and preserved with formaldehyde (final concentration 5% v/v). For microbial molecular analysis, two liters of water were collected in autoclaved HDPE bottles (Nalgene) and stored on ice. Within 8 hours, the water was vacuum-filtered onto sterile, 0.2 μm polycarbonate filters, which were transported on dry ice and stored at -80°C. Samples for cell counts and for molecular analysis were shipped to the University of Colorado at Boulder for processing.

Negative DNA controls were collected daily by filtering 2 liters of 0.22 μm filtered, distilled water collected in autoclaved Nalgene bottles. DNA extractions from the negative controls failed to amplify by PCR. A positive control was also collected by swabbing household surfaces with a pre-sterilized glove, which then was used to spike filtered, distilled water. The control served as a test for human contamination and ordination analysis demonstrated that the community did not cluster with any tap water samples.

### DNA Library Construction and Sequencing

Genomic DNA was extracted from thawed filters using a phenol:chloroform extraction with bead-beating followed by ethanol precipitation, as outlined in Holinger et al. [2]. The recovery of sufficient microbial DNA for molecular analysis differed by municipality. Two municipalities in particular, M8 and M13, had low PCR success rates. It is possible that the considerably higher aluminum (Al) concentrations present in tap waters from both municipalities, compared to other water samples, may have impeded DNA isolation or PCR amplification (S1 Table).

#### High-throughput sequencing

Of the 85 tap water samples collected, 67 yielded sufficient DNA for high-throughput sequence analysis. Bacterial profiles were determined by amplification of 16S rRNA genes and sequence analysis following previously described methods [28,29]. Amplicons were generated using primers that target the V1-V2 region (27F/338R). PCR products were normalized using agarose gel densitometry and pooled. The pooled amplicons were lyophilized, purified (Montage), concentrated using a DNA Clean and Concentrator kit (Zymo Research, USA) and adjusted to 4 nM. The pooled DNA was denatured, then diluted to 15 pM and spiked with 25% of the Illumina Phi X control DNA prior to loading the sequencer. Illumina paired-end sequencing was performed at the University of Colorado at Denver on the MiSeq platform with version v2.3.0.8 of the MiSeq Control Software and version v2.3.32 of MiSeq Reporter, using a 600-cycle v3 (2x300) reagent kit.

#### Analysis of Illumina Paired-end Reads

Paired-end sequences were sorted into paired reads [28] and deposited in GenBank under Bioproject #PRJNA255807 (accession numbers SRX698948-SRX699014). Sorted and paired reads were assembled using phrap [30,31], and pairs that did not assemble were discarded. The ends of assembled sequences were quality-trimmed over a moving window of 5 nucleotides until average quality met or exceeded 20. Trimmed sequences that were <200 nt in length or contained >1 ambiguity were also discarded. Potential chimeras were identified with Uchime [usearch6.0.203_i86linux32 [32]] using the reference sequence set of Schloss (http://www.mothur.org/wiki/Silva_reference_files) and were removed. The final sequence data set consisted of 8,648,826 sequences with an average read length of 287nt. The average sequencing depth per sample was 127,189 (min 21,712; max 428,688). Finished sequences were aligned and classified with SINA (v1.2.11, [33] using the Silva 111Ref database [34]. All unique sequences were taxonomically assigned, and operational taxonomic units (OTUs) were produced by clustering sequences with identical taxonomic assignments.

### Identification of a novel phylotype

One sample, M15S1, showed a high relative abundance of MiSeq sequences that were categorized as “Unclassified” (44%, >8,000 sequences) by SINA (http://www.arb-silva.de/aligner/). We therefore sought to resolve the taxonomy of unclassified sequences in this sample by analysis of a ~1300bp fragment of the 16S rRNA gene with the bacterial primers 8F-1391R. PCRs were set up as previously described [35] and conducted at 94°C for 2 min, followed by 30 cycles of 94°C for 20 s, 52°C for 20 s, and 65°C for 1.5 min, followed by a 65°C elongation step for 10 min. Amplicons were gel-purified (Millipore, Billerica, USA), cloned into TOPO-TA pCR4.0 vector, and transformed into electrocompetent *E*. *coli* TOP10 cells (Life Technologies, Carlsbad, USA) using the manufacturer’s protocol. Clones were sequenced by the Beckman Coulter Genomics facility following the sample submission guidelines for 96-well bacterial cultures (Danvers, USA). Bidirectional sequencing using the T3/T7 primer set was performed on an ABI PRISM 3730xl sequencer following the company’s purification and sequencing process. Unless otherwise noted, sequence assembly, end-trimming, chimera-checking and quality screening were conducted using the same methodology as the MiSeq dataset. Vector contamination was removed using CrossMatch [30,31] and Univec (http://www.ncbi.nlm.nih.gov/tools/vecscreen/univec/). Of the 84 sequences that passed the quality screening, a representative subset was deposited in GenBank with accession numbers KM972552-KM972556. These high-quality sequences were aligned with SINA and then parsimony inserted into the Silva 111 Ref guide tree with ARB [36]. Taxonomy calls for these sequences were generated by NDS export from ARB as well as via the SINA classifier per the above process for MiSeq sequences. A python script was written to compute a distance matrix for the overlapping region in all of the Sanger sequences and the 8,033 “Unclassified” MiSeq sequences in M15S1. This analysis allowed us to determine which Sanger sequences matched with the “Unclassified” MiSeq sequences. Nearly all (83/84) of the Sanger sequences matched with >97% identity to a MiSeq sequence from sample M15S1, and similar taxonomic ratios for those sequences were obtained. A single taxon belonging to the order *Thiotrichales* was a best match with the MiSeq “Unclassified” taxon. Selected Sanger sequences that phylogenetically associated with the *Thiotricales* and representative sequences from this region of the Silva guide tree were used to build a maximum likelihood, bootstrap-scored phylogenetic tree with RAxML [37] and FigTree (http://tree.bio.ed.ac.uk/software/figtree/).

### Microbial cell counts

Total cell densities were determined on preserved samples by vacuum filtration through 0.22 μm pore, black polycarbonate filters (General Electric, Trevose, PA). The filters were stained with propidium iodide (Invitrogen, Grand Island, NY), treated with Citifluor antifadent (Electron Microscopy Services, Hatfield, PA) and fixed onto microscope slides. The slides were counted on a Nikon fluorescence microscope equipped with epi-illumination at 1250x magnification, with a minimum of 15 fields per filter counted.

### Environmental Data Analysis

Data analysis was conducted on samples for which microbial sequence data were available (n = 67). Metadata on source water type, disinfectant type, and water storage methodology were obtained from the 2012 and 2013 Consumer Confidence Reports published by each municipality. The lengths of surface water stream networks were determined using the “Trace Upstream” function in *Streamer* (USGS, http://nationalmap.gov/streamer/webApp/streamer.html). Streamer is a web-based mapping application built by the USGS in cooperation with the National Atlas of the United States and the National Map, and uses digital hydrographic data for the United States at a 1:1,000,000 scale.

### Microbial community analysis

The OTU table was imported into Explicet, v2.10.5 [38] for exploration of general diversity patterns and OTU coverage. Chloroplast sequences (which generally comprised less than 5% of the total recovered sequences in a sample) were removed, followed by subsampling of the sequence data to the rarefaction point of 19,350 sequences. All analyses were carried out on the rarefied data set.

Ecological analyses of the environmental and community data were performed in R [39], with a significance level of alpha = 0.05. To explore the underlying environmental drivers of beta diversity, Permanova and Mantel tests were conducted using *Municipality* as a nested variable with the nominal variables *Source water* and *State*. Municipalities with only a single sample were removed prior to these analyses in order to reduce differences in within-group dispersion [40,41]. A similar nested approach was used to assess differences in taxon abundances by disinfection type, with a significance threshold of alpha = 0.01.

Bacterial compositions were visualized using nonmetric multidimensional scaling (NMDS), which reduces the complexity of community data into fewer dimensions, and can use any distance matrix [42]. Briefly, singleton OTUs were removed and a Bray–Curtis dissimilarity matrix was calculated. The NMDS was run using the vegan package in R [43]. A three-dimensional model produced a goodness-of-fit value of 0.12 using Kruskal’s stress formula, indicating that the ordination reasonably approximates the among-sample relationships [44]. A Shepard plot of calculated vs. raw dissimilarities showed strong nonmetric (R^2^ = 0. 987) and linear (R^2^ = 0.917) fits.

The relationships between 14 environmental parameters and bacterial composition were determined using the envfit function in the vegan package of R [43]. This method maximizes correlations between environmental vectors or factors with the ordination configuration. The lengths of the vectors represent the strength of the correlations, with significance of the fitted variables determined by permuting the environmental variables 999 times.

To determine the drivers of alpha diversity, a linear mixed effects model was created using the R package “lme”. *Municipality* was modeled as a random effect to account for the hierarchical structure of samples collected within a municipality. The restricted maximum likelihood estimation method was used to obtain an unbiased estimation of model variance. A full model containing four variables and their interactions was compared to models in which one variable was dropped sequentially, until only those parameters and interactions that significantly changed the model fit (measured using the log likelihood) remained. The final model was:

$${Sobs}_{ij}=\alpha +\;\beta_{1}xTotCl+\;\beta_{2}xWaterTemp+a_{i}+\;\varepsilon_{ij}$$

where *Sobs* is the number of observed OTUs at observation *j* in municipality *iI*; alpha represents the estimated intercept; ß_1_ and ß_2_ are the estimated slopes; *a*_i_ is the random intercept, and epsilon is the residual error. This model fit was marginally improved compared to a model containing only TotCl (P = 0.04). The model was validated by plotting the residuals against the fitted values and the parameter values as recommended in Zuur et al. [45].

Hill numbers were used to determine index-independent beta diversity, as outlined in Jost [46] using a customized R script (https://gist.github.com/darmitage/3179407). Briefly, diversity values were calculated using the equation,

$${}^{q}D={\left(\sum_{i=1}^{s}P_{i}^{q}\right)}^{\frac{1}{1-q}}$$

in which *D* is diversity at order *q* and *P* is the proportional abundance of taxon *i* for all taxa *s*. The value for order can range from 0-∞ and determines the sensitivity of *D* to rare or dominant species. For comparison, *q* = 0 produces a diversity value equal to taxon richness (rare-sensitive), *q* = 1 is equivalent to Shannon entropy, and *q* = 2 is equivalent to Simpson concentration (dominant-sensitive). As q increases, it becomes a measure of evenness [47].

## Results

### Drinking water distribution system environments

Multiple tap water samples were collected from 17 municipalities across four states (Fig 1 and S1 Table). While 15 of the municipalities disinfected exclusively with chlorine, two municipalities treated seasonally with chloramine (March-October). Principal components analysis of tap water chemistry showed that many water quality parameters clustered by municipality (S1 Fig). Arsenic (As) concentration and water temperature (WT), however, were explained by source water type (ANOVA P<0.01), with groundwater on average having higher As concentrations and lower WT than surface waters. Total cell counts varied across distribution systems from 7.3x10^2^ to 2.2x10^5^ cells/mL (S1 Table).

While no primary drinking water regulations were exceeded in the municipalities examined, several EPA secondary parameters exceeded their maximum contaminant levels (MCLs), specifically: Al (5 samples, max = 0.58 mg l^-1^, MCL = 0.2 mg l^-1^), iron (Fe, sample M11S3, 1.6 mg l^-1^, MCL = 0.3 mg l^-1^), and pH (23 samples, max = 10.23, MCL = 8.5) (S1 Table). These values may differ from those reported by the municipality due to differences in sampling locations within the DWDS. Secondary drinking water regulations establish guidelines for nuisance chemicals that do not pose known or direct health hazards, but may affect water taste and odor, and may indicate technical issues such as pipe corrosion.

For municipalities fed by surface water, nitrate (NO_3_) concentrations in tap water decreased with longitude, with higher concentrations in the eastern parts of each state (S1 Table). Nutrient data for source waters were sparse and therefore the length of the source water network was used as a proxy for drainage area, which is known to affect nutrient concentrations [48,49]. This analysis showed a strong relationship between NO_3_ and stream network length ([NO_3_] = 1.67*log10(streamlength)– 2.645, R^2^_adj_ = 0.85, S3 Fig).

### Bacterial diversity and community composition

**Fig 2:**
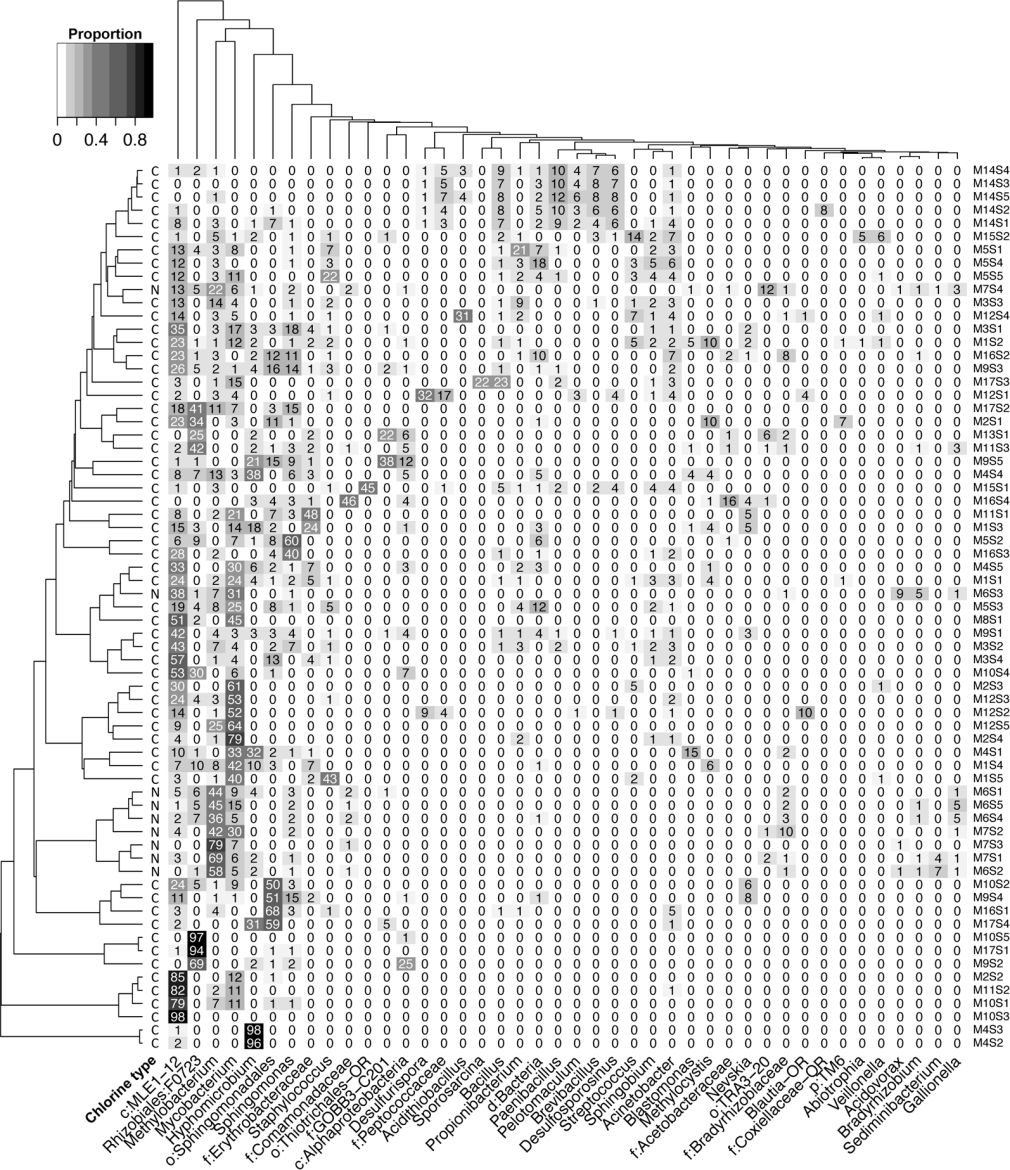
Heatmap showing distributions of bacterial taxa across tap water samples. Taxa with a minimum abundance of 5.0% in at least one sample were included. Proportional abundances are indicated with values and by shading. The dendrograms were calculated based on the complete linkage hierarchical clustering algorithm and highlight the similarities in taxonomic composition among samples (left dendrogram), and the similarities in taxon distribution (upper dendrogram). The letters denote disinfection type (C = chlorine, N = chloramine). Taxonomic identities are provided to the highest resolution provided by the Silva reference database for that OTU, typically the genus level. *f* = family; *o* = order; *c* = class; *p* = phylum. The–OR designation indicates a new taxon recovered from this study.

Sequence analysis of rRNA genes in 67 samples resulted in >8.6 million sequences, including 908 taxonomically distinct OTUs. After rarefaction, median Good’s coverage score was ≥ 99.5%, which indicates the depth of sequencing was sufficient to describe the diversity of the samples. Sample richness varied from 32 (M10S5) - 290 (M14S2) OTUs. Fig 2 summarizes the distributions of more abundant taxa among the samples, clustered to indicate the similarities among the samples; S2 Fig shows the abundance-ranked taxon distribution to 0.1% of the entire dataset, with more detailed taxonomy. Taxa representing 29 bacterial phyla were detected overall, but the distributions were highly skewed (Fig 2 and S2 Fig). Collectively, four phyla dominated the dataset: *Proteobacteria* (51% of total, 41% alpha-group), *Cyanobacteria* (18%, almost entirely MLE1-12, a non-photosynthetic member of that phylum [50]), *Actinobacteria* (16%, 13% *Mycobacterium* spp.) and *Firmicutes* (12%).

#### Bacterial distribution patterns

Several taxa showed similar distribution patterns across samples (Fig 2 and S2 Fig). For instance, *Mycobacterium* spp. and MLE1-12 cyanobacteria, the most abundant taxa overall (S2 Fig) co-occurred in 59/67 samples at abundances above 0.1% and both taxa also exhibited steep declines in abundance in samples with high sulfate concentrations (>100 mg l^-1^). The prevalence of *Mycobacterium* spp. was significantly higher in groundwater than surface water fed systems (P<0.0001), while MLE1-12 abundance did not vary significantly by source water type (P>0.05).

The relationships between taxonomic composition and environmental properties were examined using multivariate statistical analyses. A permutational MANOVA analysis (Table 1) showed, as with water chemistry, that *Municipality* (R^2^ = 0.51) and *Source Water Type (*surface vs. ground, R^2^ = 0.05) structure the overall composition of the bacterial assemblages. After controlling for *Municipality*, other factors such as type of chlorine disinfectant, tap type, and geographic location (State) did not significantly affect the total bacterial composition (Table 1).

**Table 1:** Permutational MANOVA test results of a Bray-Curtis distance matrix of bacterial communities ~ nominal variables (*n* = 65). P-value(Municipality) was calculated using Municipality as a nested variable.

	F ratio	R^2^	P-value	P-value (Municipality)
Municipality	3.66	0.506	0.001	0.001
Source Water Type	3.319	0.050	0.001	0.001
Chlorine Type	7.37	0.105	0.001	1
Fluoridation	2.299	0.035	0.011	1
State	5.25	0.205	0.001	1
Tap Type	0.910	0.029	.557	0.61

A Mantel test (Table 2) was used to weigh the influence of water chemistry and other continuous variables (S1 Table) on bacterial composition. In chlorine-treated samples, free and total chlorine concentration had the strongest relationship, while iron, nitrate, redox potential and salinity were also significantly correlated with differences in bacterial assemblages (Table 2). The sample size for chloramine treated systems was small (n = 9), however the ions Ca, K, and As were significantly correlated with bacterial assemblages (Table 2).

**Table 2:** Mantel test statistics for correlations between Bray-Curtis distances of bacterial communities and environmental parameters for chlorine and chloramine-treated samples. P-values are reported using Municipality as a nested variable. Significant P-values (<0.05) are in bold.

	Chlorine (n = 56)		Chloramine (n = 9)	
	r	P-value	r	P-value
Al	0.254	0.603	0.281	0.157
As	-0.033	0.73	0.44	**0.013**
Ba	0.173	0.806	0.2	0.585
Ca	0.117	0.586	0.378	**0.042**
Cd	-0.0349	0.73	0.319	0.142
cellCounts	0.2007	0.061	-0.011	0.704
Cr	-0.009	1	-0.298	0.981
Cu	-0.027	0.49	0.365	0.125
DO%	0.088	0.456	0.085	0.588
Fe	0.145	**0.036**	-0.164	0.888
Fl	0.156	0.421	0.203	0.591
freeCl	0.2791	**0.001**	0.246	0.146
K	0.147	0.532	0.374	**0.006**
Mg	0.13	0.298	0.361	0.224
Mn	0.14	0.098	0.102	0.387
Na	0.012	0.153	0.37	0.096
NO_3_	0.142	**0.047**	0.18	0.282
pH	0.184	0.094	-0.089	0.592
PO_4_	0.082	0.252	0.159	0.5
redoxPotential	0.153	**0.012**	-0.25	0.849
salinity	0.074	**0.034**	0.372	0.229
Sb	0.054	0.348	0.149	0.256
Se	-0.023	0.069	0.263	0.123
SO_4_	0.183	0.28	0.358	0.216
combinedCl	0.2403	**0.001**	0.102	0.333
waterTemp	0.045	0.116	-0.066	0.797
Zn	-0.059	0.665	0.334	0.097

**Fig 3:**
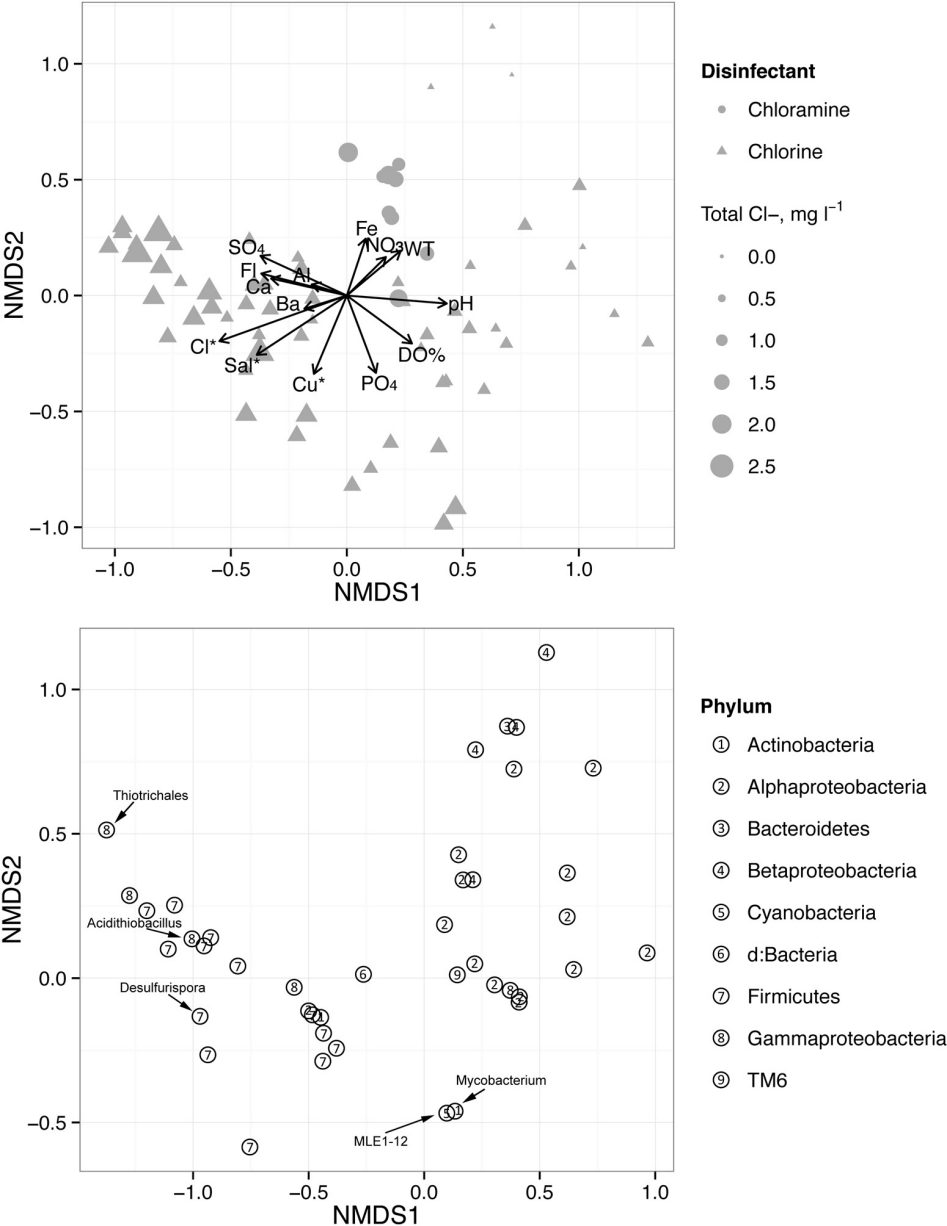
Nonmetric multidimensional scaling (NMDS) analysis of tap water bacterial assemblages (3D stress = 0.12). A. Sample coordinates plotted with fitted environmental variables. Symbols represent disinfectant and are scaled by total chlorine concentration. For the fitted environmental parameters, arrows indicate the magnitude and direction of the correlation with microbial community composition. Significantly correlated variables are noted with asterisks. B. NMDS results showing taxa with minimum relative abundances ≥ 5%. Taxa are displayed at their centroid coordinates. Numbers denote the domain, phylum or sub-phylum level classification as defined in the legend. Taxa discussed in the text are indicated.

A nonmetric multidimensional scaling (NMDS) analysis was conducted to determine the overall relationships of bacterial taxa among samples and to explore in more detail the impact of chlorination on the taxa. Environmental drivers of bacterial composition were visualized by fitting selected environmental variables to the ordination matrix, as shown in Fig 3A. In accordance with the Mantel test results, the amount of residual Cl significantly influenced bacterial composition. Bacterial assemblages from chloramine-treated samples occupied a smaller area in ordination space than chlorine-treated samples, indicating more similar bacterial communities. However, distinct clusters based on disinfection type were not observed (Fig 3). Using the same ordination results, taxa that reached abundances > 5% were plotted at their centroids in ordination space in Fig 3B. A shift in the composition of these taxa was observed with Cl concentration, with members of the alpha- and betaproteobacterial subgroups more abundant in low-Cl and Chloramine-treated systems and members of the *Firmicutes* and *Gammaproteobacteria* more abundant in higher Cl samples. The commonly co-occurring drinking water taxa *Mycobacterium* spp. and MLE1-12 occupied similar regions in ordination space (Fig 3B).

The abundances of several commonly occurring taxa were correlated with Cl concentration. Qualitatively, taxa that were more abundant in low Cl (<1.0 mg l^-1^) waters included an OTU belonging to the alphaproteobacterial group F0723 sp., which showed no significant trends with other chemical parameters. *Hyphomicrobium* spp. tended to become abundant in low Cl waters, but had much lower abundances above 1.0 mg l^-1^. Taxa belonging to the order Sphingomonadales (*Sphingomonas* spp. and others) had generally higher abundances in low and moderate (1.0–1.5 mg l^-1^) Cl waters. The OTU *Bacillus* spp. (Firmicutes), on the other hand, became more prevalent in chlorine-treated waters with moderate and high (> 1.5 mg l^-1^) Cl, high reducing potential, and relatively high ion concentrations (e.g. Ca, Al, Mg; S1 Table).

Samples with elevated SO_4_ concentrations had lower abundances of common municipal tap water taxa and higher relative abundances of putative sulfur-metabolizing taxa. For example, *Desulfurispora* spp. appeared in surface water-sourced samples at SO_4_ concentrations exceeding 150 mg l^-1^, and in groundwater-sourced samples at concentrations > 50 mg l^-1^. *Acidithiobacillus* spp., members of which commonly are found in acidic, high-S environs, were detected in three samples and dominated sample M12S4 (Figs 2 and 3). Aside from occurrence in waters with relatively low pH (7.6–8.1), no other noticeable relationships between the distribution of *Acidithiobacillus* and water chemistry were observed.

One high Cl, high SO_4_ sample (M15S1) showed a high relative abundance (44%) of “Unclassifed” sequences (“Thiotrichales-OR”, Fig 2) that had not previously been reported in DWDSs. To improve the taxonomic resolution of this novel group, near full-length 16S rRNA sequences were determined. Phylogenetic analysis (S4 Fig) assigned the sequences to the order *Thiotrichales* (*Gammaproteobacteria*), only distantly related to their nearest neighbors, with closest sequence identity (88%) to *Fangia hongkongensis* [51] and *Caedibacter taeniospiralis* [52]. These taxa form a sister group with the family *Francisellaceae*.

#### Effect of disinfectant choice on bacterial communities

**Fig 4:**
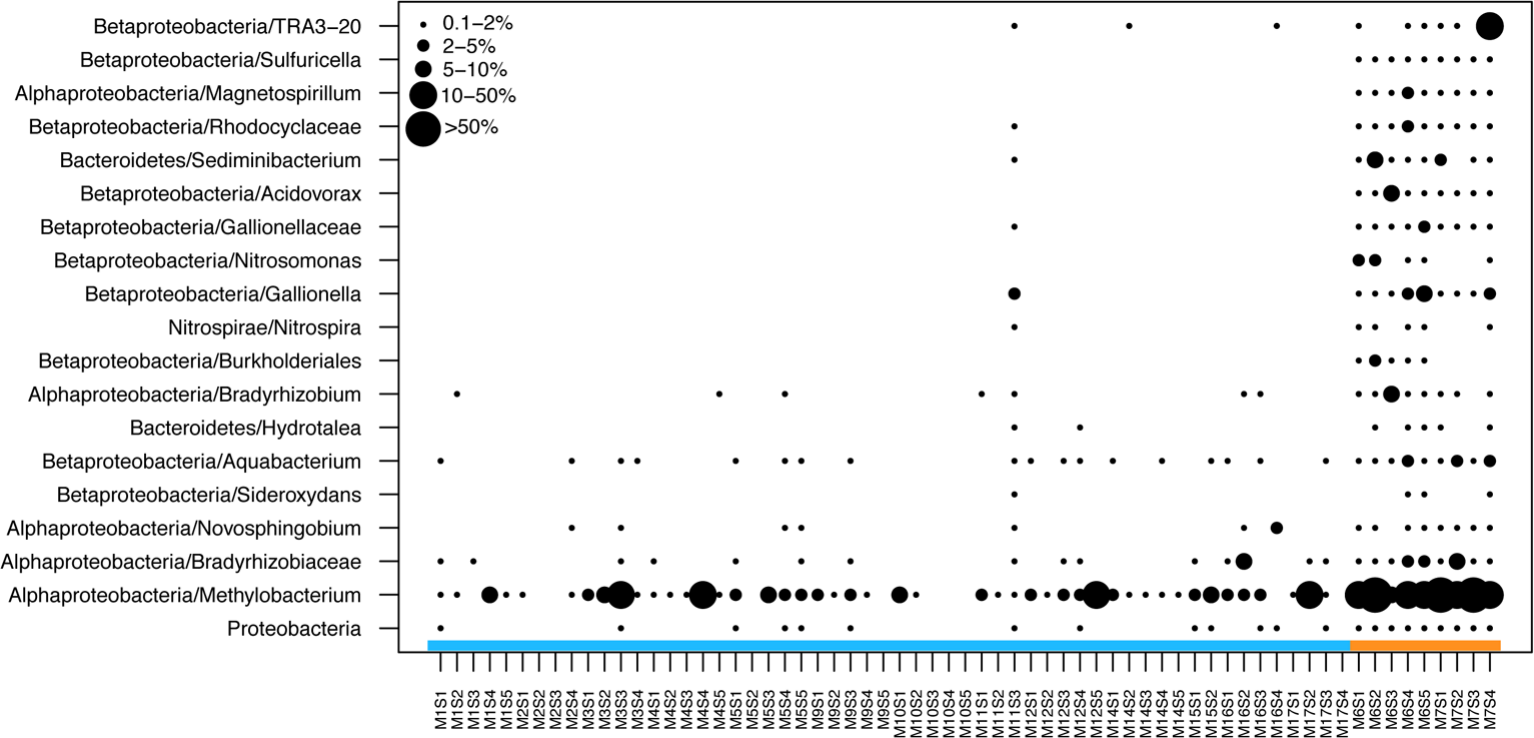
Dot plot showing the relative abundances of common (≥ 1%) bacterial OTUs that differed significantly by disinfection type (nested ANOVA, threshold P<0.01). Municipality was used as a nested variable. Colored bar along the x-axis indicates disinfectant type (blue = chlorine, orange = chloramine). Taxonomic identities are listed as phylum/genus or subphylum/genus for proteobacterial OTUs. Note the similarities between chloraminated samples and M11S3, which had exceptionally high iron.

While an effect of disinfectant type on total bacterial assemblages was not detected (Table 2), numerous taxa were significantly enriched in chloramine-disinfected systems, as determined by nested ANOVA (Fig 4). The common tap water inhabitants *Methylobacterium* spp. were highly enriched in chloramine-treated systems (Fig 4). The putative nitrifying taxa *Nitrosomonas* spp. and *Nitrospira* spp. were significantly more abundant in chloraminated systems, reaching abundances up to 3% and 1%, respectively. Chloramine-treated systems also had significantly higher abundances of several potentially metal-oxidizing taxa, similar to those found in a high-Fe, Cl-treated sample (M11S3, 1.62 mg l^-1^ compared to mean 0.037). These included members of the family *Gallionellaceae* (*Gallionella* spp. and *Sideroxydans* spp.), *Sediminibacterium* spp. and *Magnetospirillum* spp.

#### Bacterial diversity trends with total chlorine

**Fig 5:**
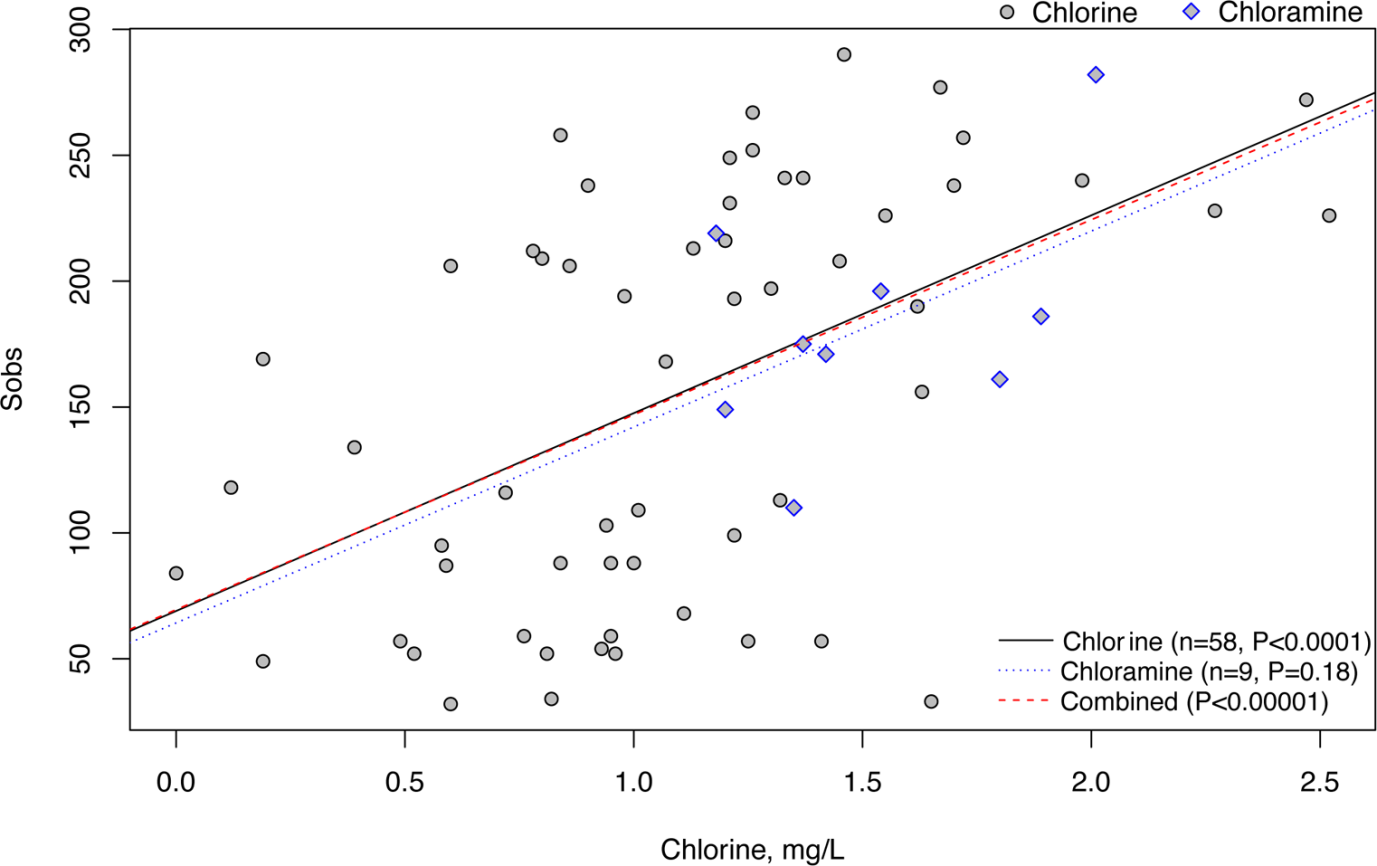
The relationship between OTU richness and total chlorine concentration. Samples from DWDSs treated with chlorine (gray circles) and chloramine (blue diamonds) were analyzed separately. Best-fit lines for each disinfectant type and for the entire dataset are shown with their respective P-values.

Over the range of Cl concentrations observed in this study (0–2.5 mg l^-1^), a positive relationship was observed between bacterial richness (Sobs) and total Cl (Fig 5). A mixed effects model that accounted for the structuring effect of Municipality found that total Cl was the single best predictor of Sobs, with a marginally significant negative effect of WT (observed vs fitted R^2^ = 0.58, P<0.0001). To determine how this relationship with Cl changed based on the relative weighting of rare and common taxa, ‘true’ diversity was calculated using Hill numbers [46,53] at different levels of total Cl (S5 Fig). Consistent with the mixed effects model results, the largest difference in diversity occurred between low and moderate-high Cl samples; high Cl samples had the highest diversity (S5 Fig). Regardless of the species weights, the mean diversity was lowest in low Cl samples.

At a broad taxonomic level, variable responses of the dominant phylotypes with chlorine concentration are summarized in S6 Fig. Consistent with the NMDS analysis (Fig 3), taxa demonstrated different sensitivities to chlorine levels. Some, for instance *Alphaproteobacteria*, were diminished at high chlorine. Others, notably *Mycobacterium* spp. (Actinobacteria), were substantially enhanced. MLE1-12 cyanobacteria were insensitive to chlorine concentration. Thus, the influence of chlorination on DWDS microbiology was evident.

## Discussion

Distribution system networks are highly heterogeneous environments and numerous factors beyond source water and treatment process can modify bacterial assemblages. Differences in pipe material [3,54], pipe age [55], the relative contribution of biofilm to bulk water [56] and distance along the distribution system network [57] can influence bacterial composition of drinking water. In this study, variability in residual chlorine concentrations within the distribution systems was evident and suggests that chlorine demand by pipe materials [58] and biofilms [59] was occurring and altered the local chemical and physical environment.

In spite of this heterogeneity, trends in the microbiology of finished waters from DWDSs in the Ohio River region were consistent with those found in other treated systems [2,4,18], with relatively low diversity [20], near-ubiquitous occurrence of the non-photosynthetic cyanobacterium MLE1-12 and *Mycobacterium* spp., and common occurrence of a few other taxa (namely alphaproteobacterial family F0723, methylobacteria, *Hyphomicrobium* spp., and sphingomonads). The results presented here therefore support the existence of a core microbiome for Cl-treated DWDSs in the U.S.

### Strength of disinfectant influences DWDS microbiology

Across the municipalities, there was a clear shift in microbial assemblages from *Alpha*- and *Betaproteobacteria* to *Gammaproteobacteria* and members of the *Firmicutes* in waters with higher residual chlorine concentrations (Fig 3 and S5 Fig). Previously, similar changes in proteobacterial populations were found in a chloramine-treated bioreactor [56] a chlorine-treated pilot DWDS [21], and an urban DWDS [20], which suggests that this pattern can occur within the same DWDS. Two hypotheses may explain this finding. The first possible explanation is differential susceptibility of taxa to oxidative stress, which suggests that ecological processes can structure DWDS microbiology. Under low Cl (i.e. low selective pressure), bacteria are structured by local environmental conditions such as concentrations of DOC, N, and P, thereby increasing the importance of resource competition and niche selection [60,61]. Under these conditions, a low-diversity community of autotrophic and heterotrophic *Proteobacteria* develops that utilizes the minimal resources available and achieves relative stability. At higher Cl, this external selection pressure becomes more important: Cl-sensitive taxa are suppressed, and Cl-insensitive *Firmicutes* and *Gammaproteobacteria* are enriched. Many known members of the *Firmicutes* produce highly resistant endospores [62] that may provide a selective advantage in relatively high-Cl systems and allow them to dominate either as aggregates or within biofilms. This hypothesis aligns with the observations of Ridgway and Olson [63] that spore-forming taxa are much more resistant to chlorine than taxa that cannot sporulate.

A second hypothesis is that the change in bacterial assemblages may result from changes in the relative contribution of biofilm to the bulk water, which was shown by Srinivasan et al. [64] to vary by Cl concentration. Biofilms were previously shown to have higher abundances of *Gammaproteobacteria*, while bulk waters had greater abundances of *Alphaproteobacteria* [23]. Mature biofilms also tend to have higher species richness [23,26], which is consistent with our finding that total chlorine concentration correlated with bacterial diversity in both chlorine and chloramine-treated DWDSs (Fig 5 and S4 Fig). A larger contribution of biofilm organisms to bulk water could result from higher oxidative stress in the bulk waters suppressing cell densities, or could reflect differences in hydraulic regime that increase biofilm scouring.

Other factors may also contribute to the observed patterns in bacterial composition and diversity. Salinity and water temperature in particular both exhibited significant relationships with bacterial composition and diversity and are known to influence microbiota in other environments. These water quality parameters are more likely to reflect source water and support previous research that source water is an important determinant of microbial composition. Nitrate also correlated with bacterial composition in chlorine-treated systems. Two possible explanations for the relationship between nitrate concentrations in tap water and geographic factors such as longitude (S1 Table) and source water (S3 Fig), are: 1) biological activity within the DWDSs was too low to significantly alter incoming nitrate concentrations; or 2) N transformation rates were consistent across the DWDSs studied here. It is also possible that observed nitrate concentrations were an artifact of treatment processes [65], and further study is needed to confirm a relationship.

### Effects of Disinfectant Choice

While we did not detect significant differences in the total bacterial assemblages due to disinfectant choice (ANOVA P = 0.449), a subset of taxa only occurred or were significantly more abundant in chloramine-treated systems than in chlorinated systems (Fig 4). We also found that seasonal chloramine use increased the abundances of putative nitrifiers and Nitrospira-like organisms (Fig 4), which were recently shown to have the capacity for complete ammonia oxidation [66]. Chloraminated DWDSs provide a source of ammonia that can alleviate N limitation for microbes, thereby promoting cell growth and nitrogen transformations. Nitrification, in which microbes convert NH_4_^+^ to NO_3_^-^, is commonly observed in chloraminated drinking water systems [13,67,68] and is known to have deleterious effects on water quality and DWDS integrity. Consequently, controlling nitrification rates is an important goal for water utilities, and is a major reason why many municipalities periodically alternate between free chlorine and chloramine disinfectants [67]. Similar to Holinger et al. [2], our results showed increased abundances of nitrifying bacteria (*Nitrosomonas* sp., Fig 4) in chloraminated systems that is consistent with the occurrence of nitrification.

Nitrification is thought to increase heterotrophic growth by increasing DOC availability [13]. Consequently, changes in the composition of heterotrophic taxa may be expected in chloraminated systems. We found that the heterotrophic, C-1 metabolizing *Methylobacterium* spp. was significantly more abundant in seasonally chloraminated systems than in chlorinated systems (Figs 3 and 5). A previous laboratory-scale study found that *Methylobacterium* spp. increased in abundance just prior to the onset of nitrification and then declined [56], prompting the authors to suggest that this taxon has nitrification potential. This interpretation contrasts with results of a study by Hwang et al [20], which showed an enrichment of *Methylobacteriaceae* during the chlorine treatment phase in a DWDS that switches seasonally between chlorine and chloramine. It should be noted that, across the entire data set, carbon may influence the overall distribution of C-1 metabolizers. For example, a previous study showed a relationship between *Methylomonas* sp. and haloacetic acids [69], and a *Methylobacterium* species has been isolated from enrichment cultures in which haloacetic acids were the sole carbon source [70]. While we did not find a significant correlation between the relative abundances of *Methylobacterium* and total carbon (results not shown), it is possible that haloacetic acids or other specific carbon contaminants contributed to the overall distributions of C-1 metabolizers.

Furthermore, we found that disinfection method may alter abundances of corrosion-promoting microbes. For example, we observed an enrichment of potentially Fe-metabolizing taxa in seasonally chloraminated systems. These taxa also reached higher relative abundances in a chlorine-treated sample that contained high Fe (M11S3), suggesting a role in Fe cycling. Similar Fe-oxidizing taxa were abundant in tap water from a DWDS in China that was experiencing a “red water” event (i.e. high iron oxides), thereby linking the microbiology with water quality [24]. These results hint that chloraminated systems may have a higher propensity for microbially induced corrosion, or at least enhanced rates of iron cycling. Other studies also have identified DNA sequences closely matching Fe-oxidizing taxa in chloraminated systems [20,71], suggesting that Fe-metabolizing microbes are widespread in such systems. Current US EPA analyses estimate that $334.8 billion will be needed between 2007 and 2026 for drinking water infrastructure [72]. Roughly 60% of that amount is required for maintaining and replacing aging distribution systems, many of which are made of iron and have known microbially induced corrosion problems [72,73].

### Novel microbial taxa in DWDSs

While most of the dominant microbial inhabitants seen in this survey have been previously identified in drinking water DWDSs, there were some exceptions. Two taxa in particular are notable. First, *Acidithiobacillus* spp. was found intermittently in M14 and at high relative abundance in M12S4, a municipality that obtains a portion of its source water from an abandoned and subsequently flooded coal mine. Members of this genus are acidophilic, known to oxidize iron [74] and sulfur compounds [75] and commonly occur in S-containing habitats such as in acid mine drainage streams [76–79] and corroding sewage pipes [80]. In the current study, the relative abundance of *Acidithiobacillus* spp. did not correspond to pH or other tap water chemistry parameters. Furthermore, the low frequency of occurrence suggests that this taxon is not a common inhabitant of DWDS’s, but could be sourced stochastically from local habitats. Such potential sources include coal mine drainages in the Appalachian basin where these municipalities reside. Abandoned and flooded coal mines are increasingly used as drinking water sources in West Virginia [81], and mines are known to influence ground and surface water in the region. Water main breaches also may provide additional entry points for this organism. Such sources may provide unique and unexpected microbiota to DWDSs.

Another previously undocumented taxon, here called Thiotrichales-OR, was found in high abundance in a single sample with high Cl concentration. The relatively large genetic distance of Thiotrichales-OR from its nearest neighbors (~12%) suggests that it belongs to a new genus or family within this order. The diversity of known bacteria of this lineage is relatively small. However, given the known diversity, Thiotrichales-OR was most closely related to taxa that have obligate intracellular lifestyles, and include the human pathogen *Francisella tularensis* [82], the fish pathogen *Piscirickettsia salmonis*, and the *Paramecium* endosymbiont *Caedibacter taeniospiralis* [52] (S4 Fig). Based on its relationship to known intracellular bacteria, our findings suggest that this taxon may live within a host that then protects it from disinfection, similar to some disease-causing bacteria that proliferate in DWDSs within eukaryotic hosts [83–85].

## Conclusions

This study focused on small to medium sized DWDSs, which have considerably higher per capita infrastructure needs than larger municipalities [72]. Considering the critical role microbes play in influencing DWDS health and longevity, understanding the microbiology of these systems may help address some of the challenges facing the nation’s infrastructure, such as to reduce capital costs, improve DWDS function, and increase the efficiency of monitoring programs. To summarize, results from this study show that: 1) As the strength of disinfectant increase, bacterial diversity also increased; 2) In chlorine-treated systems, increased disinfectant levels shifted bacterial communities from *Alphaproteobacteria* and *Betaproteobacteria* to *Firmicutes* and *Gammaproteobacteria;* 3) In chloramine-treated systems, microbes associated with nitrification and iron-cycling were prevalent; and 4) Novel taxa of potential human interest can occur. This study provides valuable baseline information for future monitoring of drinking waters that may be perturbed by, for example, changes in land use, extreme weather events, or chemical spills. While this regional survey was not designed to answer mechanistic questions, our results can be viewed in the context of theories developed in community ecology. Grounding questions about DWDS microbiology in ecological theory may provide insights into the mechanisms underlying the structure and function of microbes in these built environments.

## Supporting Information

S1 Fig Principal components analysis of selected environmental parameters. For all panels, the sizes of the ellipses are to a normal probability. A. Samples grouped by municipality. B. Samples grouped by source water type. C. Samples grouped by disinfectant type.
(TIF)

S2 Fig Heatmap of bacterial taxa with an average minimum abundance of 0.1% in the overall dataset. (PDF)

S3 Fig Relationship between tap water nitrate concentrations and stream length (a proxy for drainage area) for systems fed by surface water. R^2^ = 0.85. Tap water sourced from an open reservoir and from chloramine-treated systems are indicated.
(PDF)

S4 Fig Phylogenetic relationship between the unclassified Thiotrichales-OR (clones OR078) and related Gammaproteobacteria based on 16S rRNA gene sequences. The tree was created using the maximum likelihood method in RAxML. Numbers at the nodes represent percentages from 500 resampled datasets. Scale bar represents 0.06 nt substitutions per position.
(PDF)

S5 Fig Mean diversity values (±SE) based on Hill numbers, grouped by chlorine concentration, over a range of sensitivity (q) values. Low <1.0 mg l^-1^, Moderate 1.0–1.5, High >1.5. Inset table, significance of diversity differences at chosen values of q, with significant differences in bold. Values for pairwise comparisons were determined using the Tukey honestly significant difference test.
(PDF)

S6 Fig Mean relative abundances of dominant bacterial phyla in low (<1.0 mg l^-1^), moderate (1.0–1.5 mg l^-1^), and high (>1.5 mg l^-1^) chlorine samples. Mycobacterium and MLE1-12, of the phyla Actinobacteria and Cyanobacteria, respectively, are also shown separately to reflect their high relative abundances.
(PDF)

S1 Table Physical and chemical properties of tap water samples used in this study. NA: measurement not available; BD: value below detection limit; GW: groundwater; SW: surface water.
(CSV)
