# Supplementary figures and images for: Factors Influencing Bacterial Diversity and Community Composition in Municipal Drinking Waters in the Ohio River Basin, USA
Source: PLoS One. 2016 Jun 30;11(6):e0157966. doi: 10.1371/journal.pone.0157966 (PMC4928833; doi:10.1371/journal.pone.0157966)

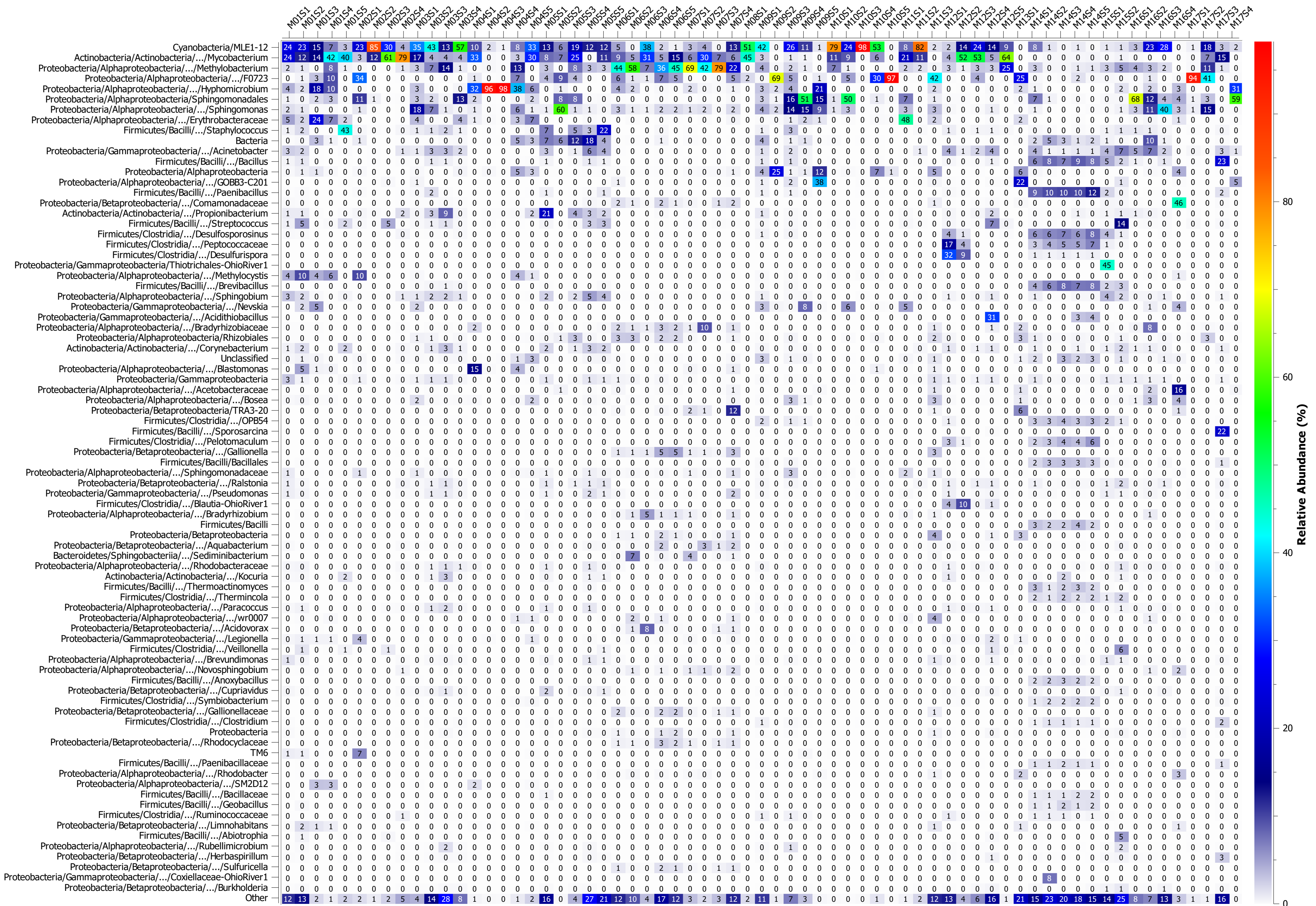

Supplement: S2 Fig — (PDF) [file pone.0157966.s002.pdf]

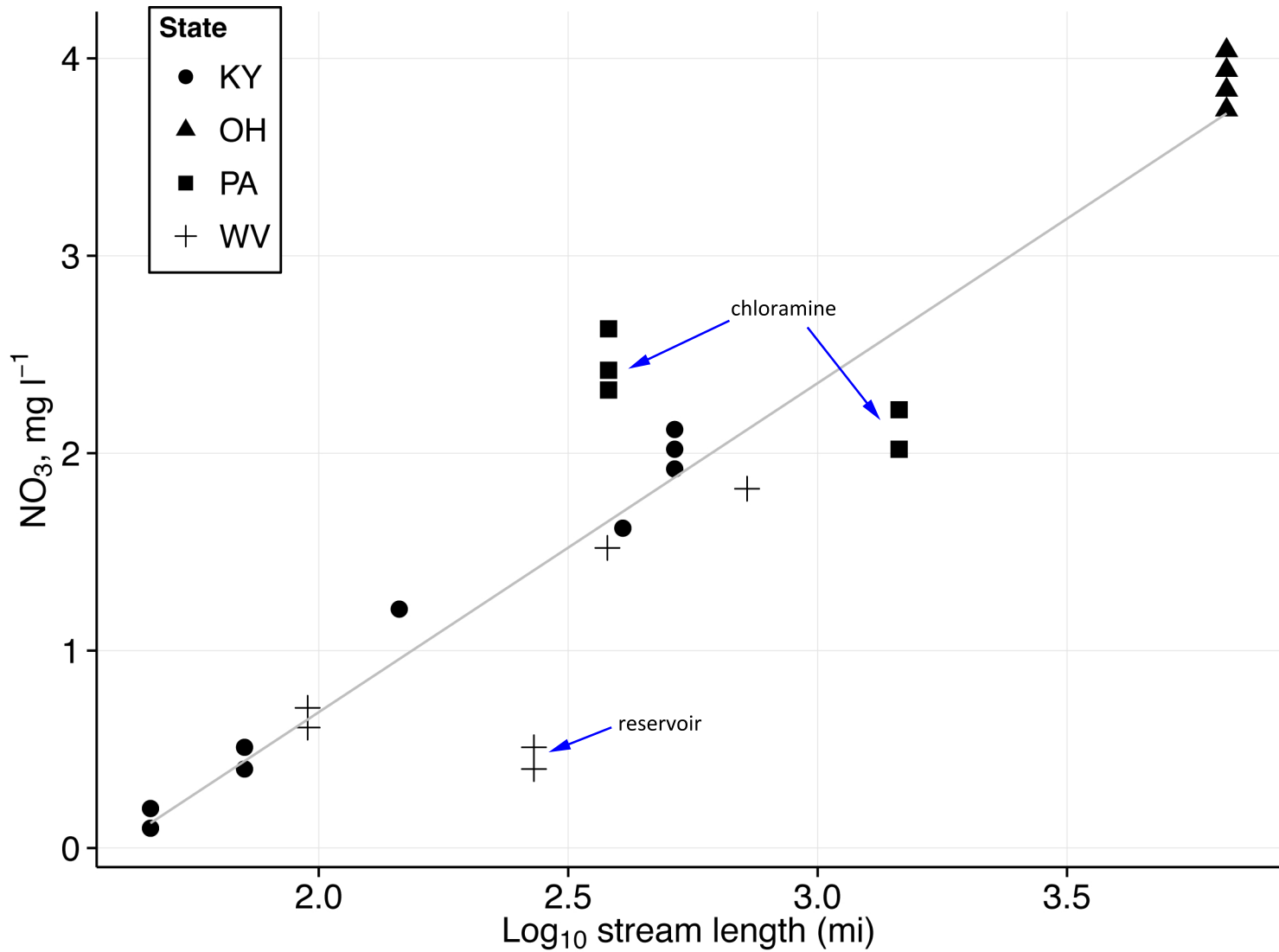

Supplement: S3 Fig — R2 = 0.85. Tap water sourced from an open reservoir and from chloramine-treated systems are indicated. (PDF) [file pone.0157966.s003.pdf]

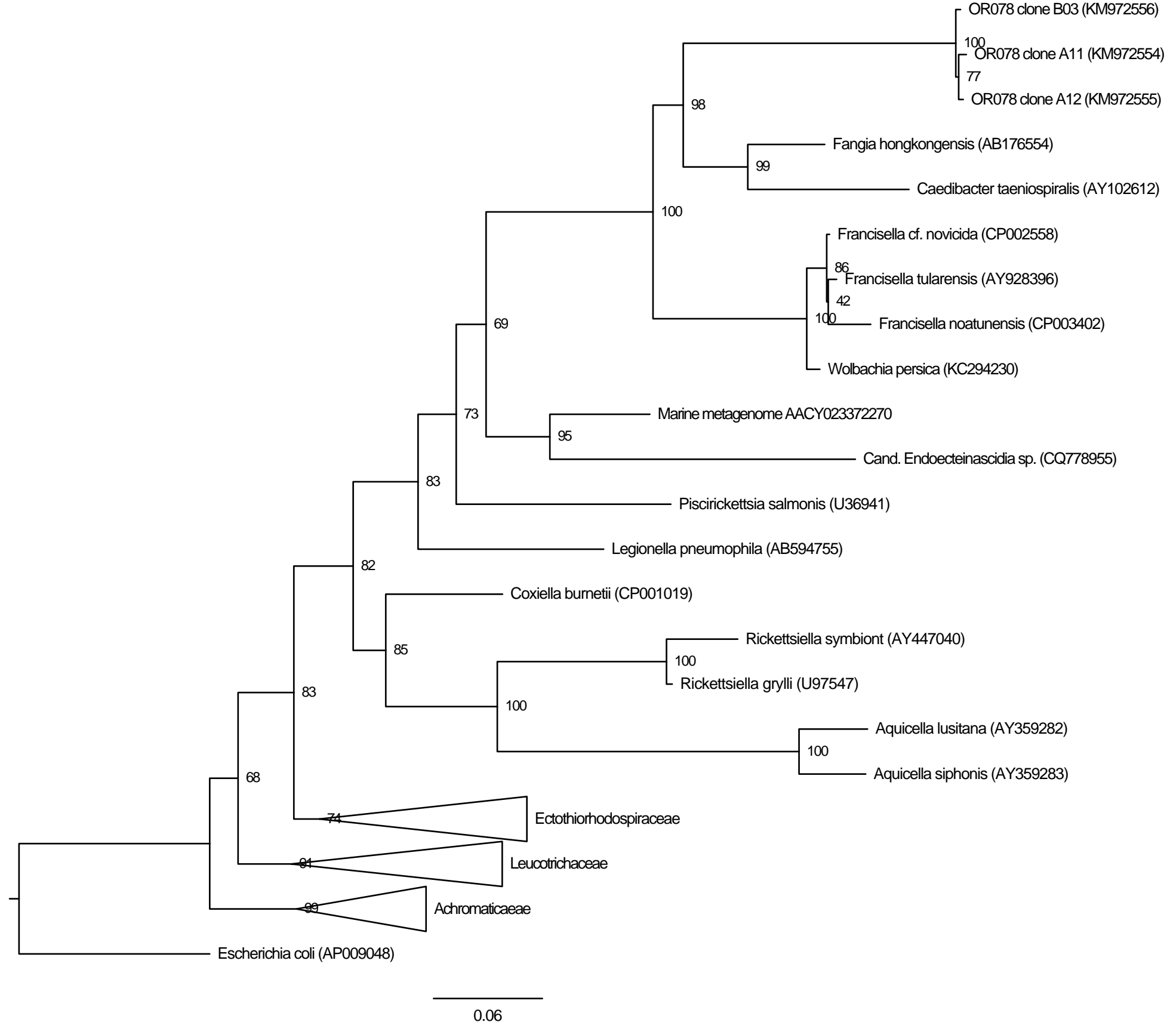

Supplement: S4 Fig — The tree was created using the maximum likelihood method in RAxML. Numbers at the nodes represent percentages from 500 resampled datasets. Scale bar represents 0.06 nt substitutions per position. (PDF) [file pone.0157966.s004.pdf]

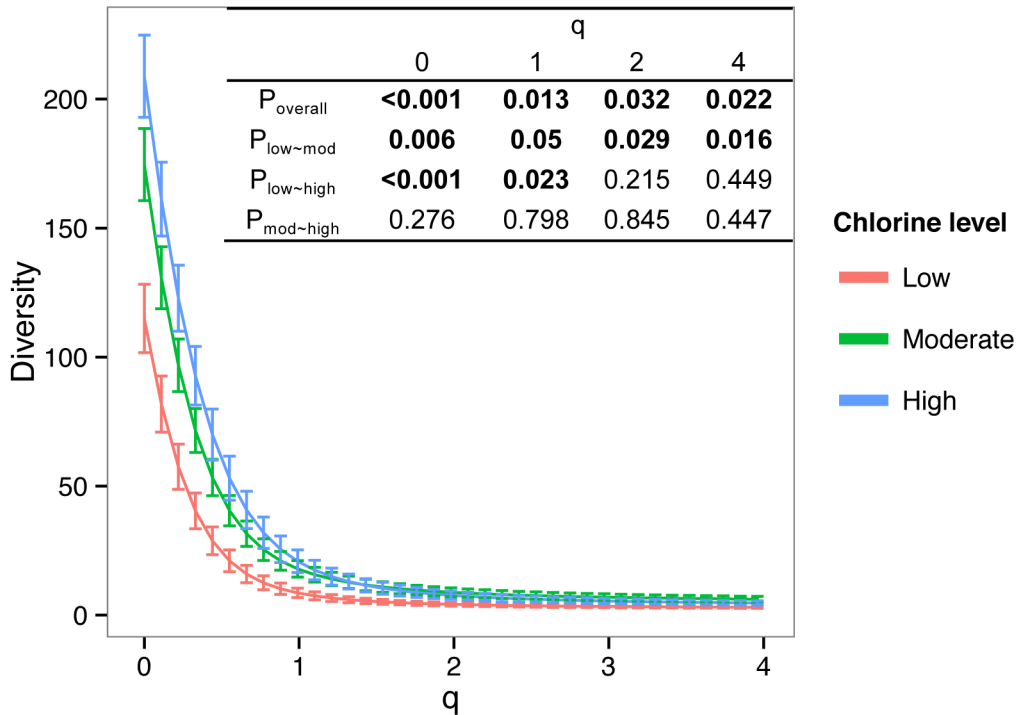

Supplement: S5 Fig — Low <1.0 mg l-1, Moderate 1.0–1.5, High >1.5. Inset table, significance of diversity differences at chosen values of q, with significant differences in bold. Values for pairwise comparisons were determined using the Tukey honestly significant difference test. (PDF) [file pone.0157966.s005.pdf]

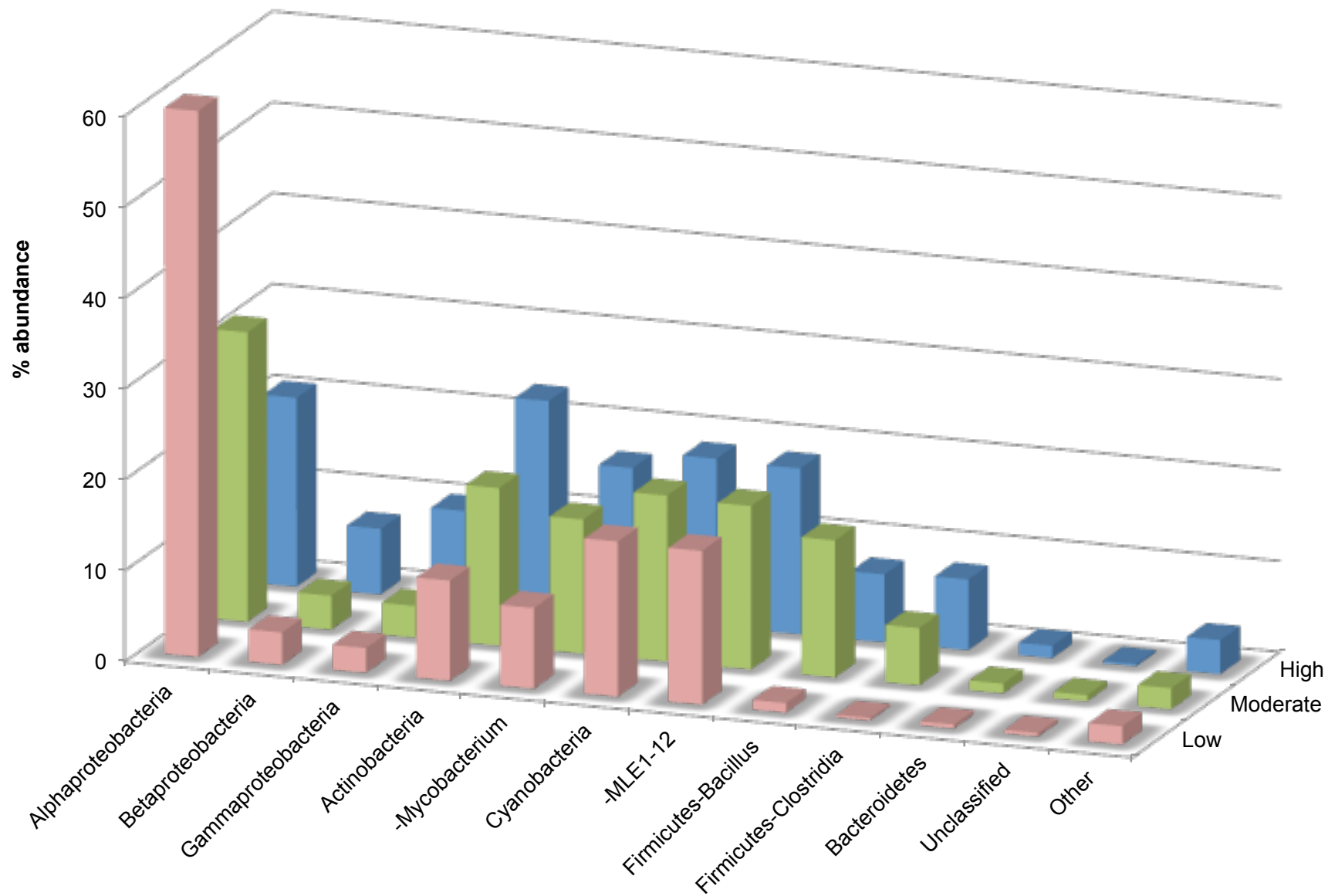

Supplement: S6 Fig — Mycobacterium and MLE1-12, of the phyla Actinobacteria and Cyanobacteria, respectively, are also shown separately to reflect their high relative abundances. (PDF) [file pone.0157966.s006.pdf]
